# Naringina e Trimetazidina Melhoram a Sensibilidade Barorreflexa e a Atividade Elétrica do Trato Solitário do Núcleo na Lesão de Isquemia-Reperfusão Renal

**DOI:** 10.36660/abc.20200121

**Published:** 2021-08-09

**Authors:** Negin Amini, Alireza Sarkaki, Mahin Dianat, Seyyed Ali Mard, Akram Ahangarpour, Mohammad Badavi

**Affiliations:** 1 Ahvaz Jundishapur University of Medical Sciences School of Medicine Department of Physiology Ahvaz Irã Department of Physiology, School of Medicine, Ahvaz Jundishapur University of Medical Sciences, Ahvaz - Irã; 2 Ahvaz Jundishapur University of Medical Sciences Research Institute of Basic Medical Sciences The Persian Gulf Physiology Research Center Ahvaz Irã The Persian Gulf Physiology Research Center, Research Institute of Basic Medical Sciences, Ahvaz Jundishapur University of Medical Sciences, Ahvaz - Irã

**Keywords:** Isquemia-Reperfusão Renal, Sensibilidade Barorreflexa, Núcleo do Trato Solitário, Naringina, Trimetazidina

## Abstract

**Fundamento:**

O núcleo do trato solitário (NTS) é uma área do cérebro que desempenha um papel fundamental na regulação renal e cardiovascular através dos impulsos dos barorreceptores.

**Objetivos:**

O objetivo deste estudo foi avaliar o efeito da Naringina (NAR) e trimetazidina (TMZ), isoladamente e combinadas, na atividade elétrica do NTS e na sensibilidade barorreflexa (SBR) na lesão de isquemia e reperfusão (I/R) renal.

**Métodos:**

Foram utilizados quarenta ratos machos Sprague-Dawley (200-250 g), alocados em 5 grupos com 8 ratos cada. Grupos: 1) *Sham*; 2) I/R; 3) TMZ 5 mg/kg; 4) NAR 100 mg/kg; e 5) TMZ5 + NAR100. A veia femoral esquerda foi canulada para infundir a solução salina ou droga e avaliar a SBR. A I/R foi induzida por oclusão dos pedículos renais por 45 min, seguida de reperfusão de 4 horas. O eletroencefalograma local do NTS foi registrado antes, durante a isquemia e durante a reperfusão. A fenilefrina foi injetada por via intravenosa para avaliar a SBR ao final do tempo de reperfusão. Os dados foram analisados por ANOVA de duas vias com medidas repetidas seguida pelo teste *post hoc* de Tukey. Um valor de p<0,05 foi considerado como significativo.

**Resultados:**

As ondas elétricas do NTS não se alteraram durante o tempo de isquemia, mas diminuíram significativamente durante todos os tempos de reperfusão. A atividade elétrica do NTS e a SBR foram reduzidas drasticamente em ratos com lesão I/R; no entanto, a administração de NAR e TMZ, isoladamente e combinadas, melhorou significativamente essas alterações em ratos com lesão I/R.

**Conclusões:**

Os resultados mostraram que a lesão de I/R leva à redução da atividade elétrica da SBR e do NTS, e pode haver uma ligação entre a I/R e a diminuição da SBR. Além disso, a NAR e a TMZ são agentes promissores para tratar complicações de I/R.

## Introdução

A lesão renal aguda (LRA) é um grande problema clínico com alta prevalência que afeta mais de 50% dos pacientes na unidade de terapia intensiva e causa mortalidade superior a 60%.^1^^,^^2^ A isquemia/reperfusão renal (I/R) é uma das causas mais importantes de LRA, e a geração de espécies reativas de oxigênio (ERO) desempenha um papel importante em eventos de lesão de I/R.^3^

A superprodução de radicais livres na lesão de I/R induz apoptose e, em última instância, morte celular e disfunção orgânica.^3^ O estresse oxidativo (ERO sobre o sistema de defesa antioxidante) é conhecido como um fator de lesão de I/R.^3^ Radicais livres de oxigênio e ERO são transmitidos pela corrente sanguínea para órgãos distantes e são considerados agentes intermediários para danos a órgãos distantes resultantes de I/R.^4^^,^^5^

O Núcleo do Trato Solitário (NTS) atua como a porta de entrada para o sistema nervoso central para inserir informações sensoriais que desempenham um papel importante na regulação cardiovascular.^6^ Barorreceptores periféricos, quimiorreceptores e nervos aferentes simpáticos renais criam a sinapse primária no NTS.^7^ A disfunção dos barorreceptores leva à perda da regulação das flutuações da pressão arterial e diminuição da sensibilidade barorreflexa (SBR), a qual é uma base fisiopatológica bem conhecida nos distúrbios cardiovasculares.^8^ As evidências disponíveis indicam que a degradação do NTS leva a alternâncias da pressão arterial.^7^ Portanto, o NTS é um dos principais centros de regulação da SBR.^8^

A Naringina (4, 5, 7-trihidroxiflavanona-7-ramnoglucosídeo, NAR) é um composto polifenol encontrado principalmente na toranja e em várias plantas cítricas. Os efeitos antimicrobianos, antimutagênicos, anticâncer, antiinflamatórios, eliminadores de radicais livres e antioxidantes da NAR foram demonstrados.^9^^,^^10^ Os efeitos protetores da NAR através do aumento da atividade de enzimas antioxidantes foram documentados.^9^^,^^10^

A trimetazidina (dicloridrato de 1- [2,3,4-trimetoxibenzil] piperazina, TMZ), é uma droga anti-isquêmica utilizada na angina instável.^11^ O poro de transição da permeabilidade mitocondrial (mPTP, do inglês *mitochondrial permeability transition pore*) localizado na membrana interna da mitocôndria desempenha um papel poderoso na produção de ERO e no início da apoptose após lesão de I/R.^12^ Um estudo experimental documentou que a TMZ foi capaz de inibir o mPTP e reduzir o tamanho do infarto na lesão de I/R do miocárdio e a atividade da caspase-3.^13^ Além disso, foi relatada a inibição da peroxidação lipídica pela TMZ.^14^ O objetivo deste estudo foi avaliar o efeito da NAR e TMZ, isoladamente e em combinação, na atividade elétrica de campo local do NTS, no eletroencefalograma (EEG) local e na SBR após lesão renal de I/R.

## Métodos

### Drogas

TMZ, NAR, cetamina, xilazina e uretano foram adquiridos da Sigma Co. USA. NAR e TMZ foram dissolvidas em água destilada, e o uretano foi dissolvido em solução salina normal imediatamente antes do uso.

### Animais

No estudo atual, e de acordo com nossos estudos anteriores e outros semelhantes, quarenta ratos machos Sprague-Dawley (pesando 200-250 g) foram adquiridos no centro de criação e cuidados de animais da Ahvaz Jundishapur University of Medical Sciences (AJUMS). Todos os animais foram alojados em gaiolas padrão (4 em cada gaiola) sob temperatura (22 ± 2°C) e umidade (50-55%) controladas e ciclo claro/escuro de 12 h (luzes ligadas às 07:00 da manhã), com livre acesso a *pellets* de ração alimentar e água da torneira. Os ratos foram alocados de forma simples e não randomizada em cinco grupos com oito ratos cada. Grupos: 1) *Sham*, 2) I/R, 3) TMZ 5 mg/kg; ratos com lesão de I/R receberam TMZ (5 mg/kg, iv) cinco minutos antes da reperfusão,^15^ 4) NAR 100 mg/kg; ratos com lesão de I/R receberam NAR (100 mg/kg, ip) uma vez por dia durante sete dias antes da I/R,^10^ e 5) TMZ 5 mg/kg + NAR 100 mg/kg; ratos com lesão de I/R receberam TMZ 5 mg/kg + NAR 100 mg/kg. Os ratos nos grupos *sham* e I/R receberam veículo (solução salina estéril). Os ratos do grupo *sham* foram submetidos a procedimento cirúrgico idêntico, sem clampeamento e indução de I/R.

### Cirurgia estereotáxica para implante de eletrodo

Uma semana antes do registro do EEG, os ratos foram anestesiados com cetamina (50 mg/kg) e xilazina (5 mg/kg), por via intraperitoneal. A temperatura corporal dos ratos foi mantida em 36,5 ± 0,5°C utilizando almofadas térmicas com as cabeças montadas em um dispositivo estereotáxico (Narishige Co., Japão) para a cirurgia de implantação de eletrodos. Um eletrodo bipolar de fio aço inoxidável revestido em teflon (0,005” tipo “*bare*”, 0,008” revestido, A-M Systems, Inc. WA) foi implantado no NTS com o atlas estereotáxico de Paxinos e Watson com coordenação de AP = −14,04 mm até o bregma; ML = 0,4 mm e DV = 8 mm a partir da dura-máter, de maneira adequada.^16^ Todos os implantes foram fixados no crânio com resina acrílica odontológica e dois pequenos parafusos-âncora de vidro.

### Indução de isquemia/reperfusão (I/R) renal

Os ratos foram mantidos em jejum durante a noite antes da cirurgia (por pelo menos 10 h), mas com livre acesso à água. No dia da cirurgia, os ratos de cada grupo foram anestesiados com uretano (1,7 g/kg, i.p.).^17^ Em seguida, os ratos foram colocados sobre uma almofada térmica (Harvard Apparatus, Reino Unido) para manter a temperatura corporal em aproximadamente 37 ºC. Quinze minutos após a anestesia, a veia femoral esquerda foi cateterizada utilizando um cateter de polietileno (PE50) para infusão de solução salina ou TMZ, e a artéria femoral esquerda foi utilizada para medir a pressão arterial e a SBR. Os rins esquerdo e direito foram expostos através de uma incisão na linha média. A isquemia bilateral foi induzida por oclusão de ambos os pedículos renais através de clampeamento não-traumático por 45 min. Depois disso, as pinças de clampeamento foram removidas e a reperfusão continuou por 4 horas.^18^

### Registro local de EEG

Potenciais de campo locais (EEG local) do NTS dos ratos foram adicionados a um bio-amplificador ML135 (aquisição de dados de 4 canais, Power Lab. e software Lab Chart versão 7, AD Instruments Co., Austrália) com amplificação de 1 mV, taxa de amostragem de 400 Hz e filtragem de passa-banda de 0,3–70 Hz por 5 minutos. O período de variações básicas de 5 segundos do EEG foi comparado em todos os grupos. A potência elétrica das bandas de frequência foi medida em mV^2^/Hz. O registro do EEG local foi realizado antes da isquemia por 45 min e durante o tempo de isquemia e reperfusão, de maneira adequada.

### Medida da pressão arterial média

A pressão arterial média foi registrada através da cateterização da artéria femoral esquerda conectada a transdutor de pressão e monitorada através do Power Lab System (AD Instruments, Austrália), antes da isquemia por 20 min para adaptação, durante o tempo de isquemia e reperfusão.

### Sensibilidade barorreflexa

Em todos os grupos, ao final do período de reperfusão, foram realizadas injeções intravenosas de fenilefrina (10 a 20 µg/kg) e as alterações na pressão arterial e frequência cardíaca foram registradas com transdutor de pressão e monitoradas e registradas em um PC, utilizando o software Lab Chart. Intervalos de 15 minutos de recuperação foram realizados entre as injeções da droga para atingir o nível anterior de pressão arterial. Para cada injeção, a amplitude máxima da pressão resultante e bradicardia foram utilizadas para calcular a pressão arterial média (ΔPAM) e as alterações da frequência cardíaca (ΔFC). A razão da variação de ΔFC para a variação de ΔPAM foi utilizada como o índice da SBR.^19^

### Análise estatística

Os dados obtidos para pressão arterial média, frequência cardíaca e EEG local foram analisados com ANOVA de duas vias com medidas repetidas seguida pelo teste *post hoc* de Tukey para comparações múltiplas utilizando o software Prisma versão 6.0 (San Diego, CA). Os dados foram expressos como média e desvio padrão (DP). Valores de p <0,05 foram considerados como diferenças significativas.

## Resultados

### Efeito da NAR, TMZ ou a combinação de ambas na atividade elétrica de campo local do NTS

Não houve alterações significativas na potência elétrica do NTS durante o período pré-I/R e durante os 45 minutos de isquemia. Entretanto, a potência elétrica do NTS diminuiu drasticamente durante toda a 1ᵃ, 2ᵃ, 3ᵃ e 4ᵃ horas do período de reperfusão no grupo I/R em comparação com os ratos do grupo *sham*. Por outro lado, a administração de NAR e TMZ, isoladamente ou combinadas, melhorou sua energia em comparação com o grupo I/R (Tabela 1).

**Tabela 1:** Efeito do pré-tratamento com Naringina (NAR), Trimetazidina (TMZ) ou combinação de ambas na atividade elétrica do NTS após lesão renal de I/R

Grupos	Potência do EEG (mV2/Hz)					
	Pré-IR	Isquemia	Tempo de reperfusão (h)			
			1º	2º	3º	4º
Sham	0,723 ± 0,117	0,675 ± 0,126	0,699 ± 0,116	0,68 ± 0,104	0,673 ± 0,104	0,695 ± 0,13
IR	0,725 ± 0,081	0,652 ± 0,052	0,635 ± 0,095***	0,61 ± 0,082***	0,587 ± 0,042***	0,592 ± 0,025***
NAR	0,705 ± 0,034	0,653 ± 0,051	0,673 ± 0,029	0,65 ± 0,033#	0,63 ± 0,041#	0,646 ± 0,038##
TMZ	0,712 ± 0,067	0,668 ± 0,085	0,673 ± 0,073	0,65 ± 0,069#	0,632 ± 0,054#	0,637 ± 0,069#
NAR+TMZ	0,75 ± 0,07	0,65 ± 0,069	0,673 ± 0,064	0,65 ± 0,047#	0,627 ± 0,049#	0,64 ± 0,053##

Os dados foram representados como média ± DP (n = 8). Sham (grupo cirúrgico sham), I/R (Isquemia-reperfusão + solução salina normal), NAR (I/R + NAR 100 mg/kg, ip, por uma semana), TMZ (I/R + TMZ 5 mg/kg, iv, antes da reperfusão). NAR + TMZ, combinação de NAR e TMZ. ANOVA de duas vias com medidas repetidas seguida pelo teste post hoc de Tukey.

*** p<0.001, vs. grupo sham.

# p<0.05

## p<0.01, vs. grupo I/R.

### Efeito da NAR e TMZ na frequência cardíaca e pressão arterial

**Figura 1:**
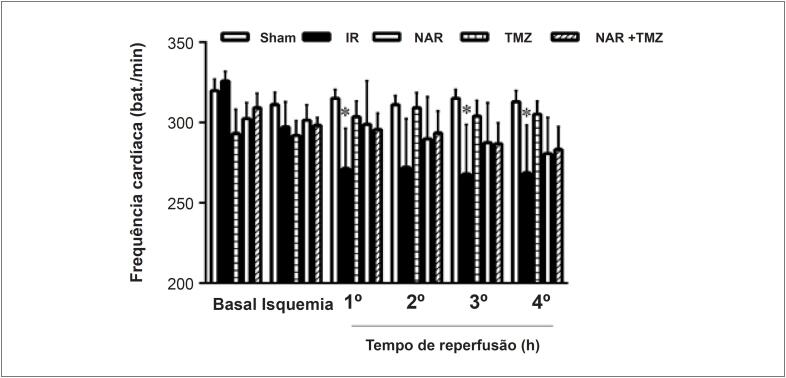
Efeito do pré-tratamento com Naringina (NAR), Trimetazidina (TMZ) ou a combinação de ambas na frequência cardíaca após lesão de I/R renal (I/R). Os dados foram representados como média ± DP (n = 8). Sham (grupo cirúrgico sham), I/R (Isquemia-reperfusão + solução salina normal), NAR (I/R + NAR 100 mg/kg, ip, por uma semana), TMZ (I/R + TMZ 5 mg/kg, iv, antes da reperfusão). NAR + TMZ, combinação de NAR e TMZ. ANOVA de duas vias com medidas repetidas seguida pelo teste post hoc de Tukey. *p<0,05, vs. grupo sham.

**Figura 2:**
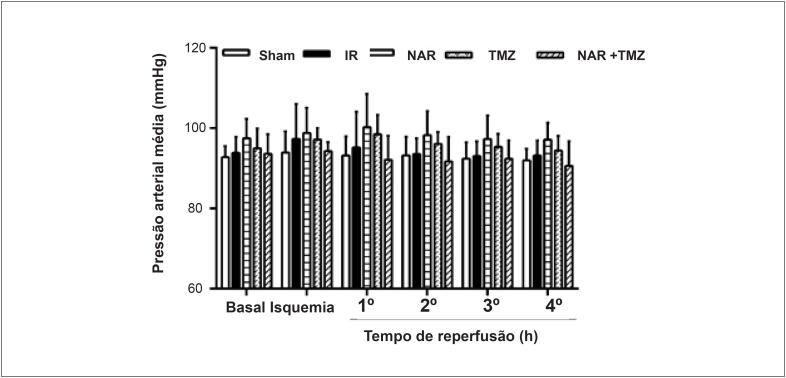
Efeito da Naringina (NAR), Trimetazidina (TMZ) ou a combinação de ambas na pressão arterial média após lesão de I/R renal (I/R). Os dados foram representados como média ± DP (n = 8). Sham (grupo cirúrgico sham), I/R (Isquemia-reperfusão + solução salina normal), NAR (I/R + NAR 100 mg/kg, ip, por uma semana), TMZ (I/R + TMZ 5 mg/kg, iv, antes da reperfusão). NAR + TMZ, combinação de NAR e TMZ. ANOVA de duas vias com medidas repetidas seguida pelo teste post hoc de Tukey.

A lesão renal de I/R reduziu significativamente a frequência cardíaca, enquanto o pré-tratamento com NAR, TMZ ou a combinação de ambas restaurou de alguma forma a frequência cardíaca aos valores normais (Figura 1). Em relação à pressão arterial média, os resultados não mostraram diferenças entre os grupos (Figura 2).

### Efeito de NAR e TMZ na SBR

Como mostrado na 3, a SBR foi significativamente reduzida no grupo com lesão de I/R quando comparado ao grupo *sham*. No entanto, a administração de NAR ou TMZ restaurou-a; portanto, não houve diferença entre os grupos *sham*, NAR e TMZ; contudo, a combinação de ambas elevou a SBR de forma mais significativa.

**Figura 3:**
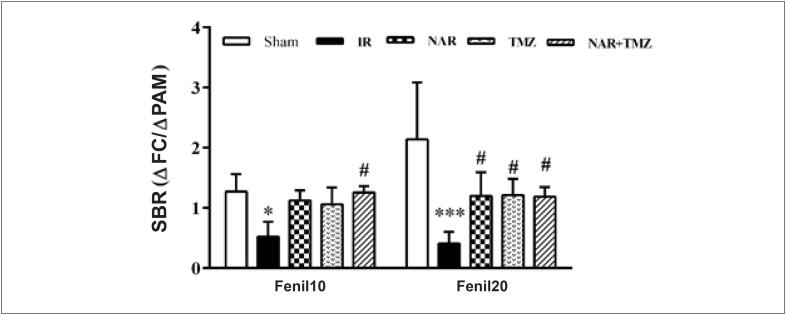
Efeito do pré-tratamento com Naringina (NAR), Trimetazidina (TMZ) ou a combinação de ambas na sensibilidade barorreflexa (ΔFC/ΔPAM) após I/R renal (I/R). Os dados foram representados como média ± DP (n = 8). Sham (grupo cirúrgico sham), I/R (Isquemia-reperfusão + solução salina normal), NAR (I/R + NAR 100 mg/kg, ip, por uma semana), TMZ (I/R + TMZ 5 mg/kg, iv, antes da reperfusão). NAR + TMZ, combinação de NAR e TMZ. ANOVA de duas vias com medidas repetidas seguida pelo teste post hoc de Tukey. *p<0.05, ***p<0.001, vs. grupo sham, #p< 0.05 vs. grupo I/R.

## Discussão

Os achados do presente estudo demonstraram que a lesão de I/R enfraqueceu a atividade elétrica do NTS e da SBR. Entretanto, as mudanças nas ondas cerebrais após a I/R mostraram que a atividade elétrica do NTS foi suprimida e essas alterações foram afetadas por uma possível disfunção cardíaca e renal. A atividade elétrica anormal subsequente do NTS pode prejudicar ainda mais a função cardíaca e agravar as complicações isquêmicas na função renal. Por outro lado, o pré-tratamento com NAR e TMZ por si só restaura as potências elétricas gama e delta, enquanto sua combinação com TMZ melhora todas as outras ondas elétricas registradas do NTS e também restaura a SBR. As fibras aferentes dos barorreceptores são recebidas principalmente no NTS, que tem uma complexa relação neuronal com outras áreas do sistema nervoso central e área vasomotora (o núcleo ambíguo e a medula ventrolateral rostral).^20^ Vários estudos já mostraram que doenças como o diabetes, a hipertensão e a insuficiência renal mediada por estresse oxidativo podem enfraquecer a SBR.^21^ A LRA aumenta a produção de citocinas anti-inflamatórias e reduz a *clearance* de citocinas, levando assim à respostas inflamatórias sistêmicas aumentadas.^22^^,^^23^ Um estudo anterior mostrou uma correlação relativa entre a disfunção dos barorreceptores e o estresse oxidativo.^24^ Outros estudos mostraram que os antioxidantes podem melhorar a SBR em vários modelos experimentais.^25^ Por outro lado, verificou-se que a administração de sequestrantes de radicais livres como a enzima superóxido dismutase (SOD) e a catalase (CAT) em coelhos com aterosclerose foi capaz de melhorar a função dos barorreceptores, indicando a inibição do efeito das ERO no desempenho dos barorreceptores.^26^

No presente estudo, a NAR e a TMZ, isoladamente ou combinadas, melhoraram a SBR, que pode ter ocorrido através de seus efeitos antioxidantes, através do sequestro da peroxidação lipídica. Nosso estudo anterior mostrou que a NAR ou a TMZ ou a combinação de ambas pode reduzir a disfunção glomerular através do aumento da capacidade antioxidante e redução do nível de microRNA-10a.^27^ Em concordância com este estudo e outros achados anteriores, a SBR é atenuada pelo estresse oxidativo, e os compostos polifenóis a melhoram através do sequestro dos radicais livres.^28^ Além disso, muitas evidências indicaram que a lesão de I/R levou à redução da síntese de óxido nítrico, que é um dos principais contribuintes de disfunção endotelial, seguida pela disfunção dos barorreceptores.^29^ A este respeito, a NAR melhora a disfunção endotelial sintetizando e aumentando a biodisponibilidade do óxido nítrico.^30^ Em um estudo realizado em humanos, também foi demonstrado que a TMZ melhorou a disfunção endotelial na insuficiência cardíaca crônica, ao reduzir os níveis de peroxidação de lipídios.^31^ Um estudo experimental mostrou que a TMZ reduziu o malondialdeído, um índice de lesão oxidativa renal, em um modelo de lesão de I/R renal.^14^ A TMZ estimula a oxidação da glicose ao reduzir a oxidação dos ácidos graxos beta, que levam à produção de ATP com menor consumo de oxigênio.^11^

Os resultados deste estudo mostraram a melhora da SBR com o pré-tratamento com NAR e/ou TMZ na I/R. Embora o mecanismo preciso dos efeitos antioxidantes de NAR e TMZ na SBR não esteja claro na I/R renal, é possível que a NAR, a TMZ ou a combinação de ambas aumentem a SBR na I/R renal devido à melhora da função do sistema nervoso autônomo. Recentemente, foi demonstrado que a administração de antioxidantes pode melhorar a SBR através da melhora da função autonômica.^24^ Os mecanismos centrais da NAR e TMZ precisam ser esclarecidos em novos estudos sobre a função do sistema nervoso autônomo.^25^

A redução da função renal leva ao acúmulo de toxinas e ao aumento da osmolalidade sérica, o que pode estimular diretamente a síntese do fator de crescimento endotelial vascular. Além disso, o aumento da produção de ERO leva ao dano endotelial e à permeabilidade da barreira hematoencefálica (BHE).^32^^,^^33^ Várias linhas de estudos mostraram que o desempenho da BHE foi interrompido em modelos experimentais de LRA indicados pelo corante azul de Evans sobre a permeabilidade para o tecido cerebral.^34^^,^^35^ Por outro lado, modelos inflamatórios experimentais induzidos pelo fator de necrose tumoral alfa (TNF-α) aumentam a permeabilidade da BHE de forma anormal. Esses estudos experimentais apoiam a ideia de que a inflamação associada à LRA aumenta as citocinas inflamatórias na corrente sanguínea e isso pode prejudicar a permeabilidade da BHE.^36^ Muitas evidências demonstraram que a NAR é um potente antioxidante que atravessa a BHE e reduz os fatores inflamatórios para proteger o cérebro.^37^^,^^38^ Uma queda abrupta na função renal leva ao acúmulo de toxinas e ao aumento da osmolalidade sérica, o que pode aumentar as ERO, resultando em lesão endotelial, ruptura da BHE e dos transmissores cerebrais.^39^

Um estudo experimental mostrou que a lesão cerebral de I/R resulta em alterações nos parâmetros eletrofisiológicos cardíacos, bem como na redução da atividade elétrica do NTS. Entretanto, a administração de antioxidantes evitou essas complicações.^16^ A redução da disponibilidade de oxigênio para o sistema neuronal, seguida pela ruptura dos vasos sanguíneos levou a uma cascata de eventos, incluindo ativação de receptores de glutamato e influxo de Ca^2 +^.^40^ A ativação dos receptores de glutamato causou um aumento na concentração citoplasmática de Ca^2+^ como resultado do influxo de Ca^2+^ através dos canais dos receptores α-amino-3-hidroxi-5-metilisoxazol-4-propionato (AMPA) e N-metil-D-aspartato (NMDA) e canais de Ca^2+^ dependentes de voltagem (VDCC, do inglês *voltage-dependent Ca2+ channels*). A abertura dos receptores inotrópicos de glutamato e o consequente influxo de Na^+^ e Ca^2+^ são identificados como os primeiros estágios do processo excitotóxico.^40^

Outro estudo experimental mostrou que LRA induzida por I/R renal levava à disfunção renal e aumento da atividade do nervo simpático renal e aumento das concentrações de norepinefrina, indicando o papel do sistema nervoso simpático no desenvolvimento da LRA.^41^ Recentemente, o papel dos nervos renais no modelo de I/R renal mostrou que a denervação do nervo renal simpático melhorou a função do órgão, reduziu a resposta dos fatores inflamatórios e a apoptose sem alterações na pressão arterial.^42^ Por outro lado, Mitaka et al. mostraram que a I/R renal levou a uma redução da pressão arterial e nenhuma alteração da frequência cardíaca, o que se opõe aos resultados deste estudo.^43^ Essa controvérsia pode estar relacionada ao modelo experimental, à espécie animal e ao período de reperfusão. Nosso estudo anterior indicou que a lesão renal de I/R resulta em disfunção renal e lesão miocárdica, e o pré-tratamento com NAR e TMZ, isoladamente ou combinadas, poderia ter papel protetor no efeito remoto da LRA no estresse oxidativo e lesão miocárdica através da regulação do Nrf-2.^44^

### Limitações

Este estudo apresentou algumas limitações. Primeiramente, ele é parte de uma tese de doutorado, incluindo limitações financeiras e de tempo. Portanto, não foi possível identificar alguns parâmetros como histologia cerebral e medidas de parâmetros moleculares e antioxidantes no tecido cerebral. Nosso objetivo foi investigar a atividade da SBR e do NTS no modelo de LRA.

## Conclusão

Nossos achados, juntamente com os achados de outras pesquisas, estão de acordo com o presente estudo, que sugere que a redução da função renal devido à lesão de I/R renal leva à redução da atividade elétrica da SBR e do NTS. Provavelmente, existe uma ligação entre a redução da função renal e a diminuição da SBR, embora a NAR e a TMZ, isoladamente ou combinadas, melhorem a atividade elétrica da SBR e do NTS. Entretanto, pode-se esperar que esses tipos de agentes antioxidantes possam ser utilizados para prevenir complicações renais de lesão de I/R em áreas além do local da lesão.
