# Supplementary material for: Naringina e Trimetazidina Melhoram a Sensibilidade Barorreflexa e a Atividade Elétrica do Trato Solitário do Núcleo na Lesão de Isquemia-Reperfusão Renal
Source: Arq Bras Cardiol. 2021 Aug 9;117(2):290–7. [Article in Portuguese] doi: 10.36660/abc.20200121 (PMC8395798; doi:10.36660/abc.20200121)
Supplement: Translation

# Translation: Naringin and Trimetazidine Improve Baroreflex Sensitivity and Nucleus Tractus Solitarius Electrical Activity in Renal Ischemia-Reperfusion Injury

**Authors:** Negin Amini, Alireza Sarkaki, Mahin Dianat, Seyyed Ali Mard, Akram Ahangarpour, Mohammad Badavi

**Affiliations:** 1 Ahvaz Iran Department of Physiology, School of Medicine, Ahvaz Jundishapur University of Medical Sciences, Ahvaz - Iran; 2 Ahvaz Iran The Persian Gulf Physiology Research Center, Research Institute of Basic Medical Sciences, Ahvaz Jundishapur University of Medical Sciences, Ahvaz - Iran

**Keywords:** Renal ischemia-reperfusion injury, Baroreflex sensitivity, Nucleus tractus solitaries, Naringin, Trimetazidine

## Abstract

**Background:**

Nucleus tractus solitarius (NTS) is a brain area that plays a key role in kidney and cardiovascular regulation via baroreceptors impulses.

**Objectives:**

The aim of this study was to evaluate the effect of naringin (NAR) and trimetazidine (TMZ) alone and their combination on NTS electrical activity and baroreceptor sensitivity (BRS) in renal ischemia- reperfusion (I/R) injury.

**Methods:**

Forty male Sprague-Dawley rats (200- 250 g) were allocated into 5 groups with 8 in each. 1) Sham; 2) I/R; 3) TMZ 5 mg/kg; 4) NAR 100 mg/kg; and 5) TMZ5+ NAR100. The left femoral vein was cannulated to infuse saline solution or drug and the BRS was evaluated. I/R was induced by occlusion of renal pedicles for 45 min, followed by 4 hours of reperfusion. The NTS local electroencephalogram (EEG) was recorded before, during ischemia and throughout the reperfusion. Phenylephrine was injected intravenously to evaluate BRS at the end of reperfusion time. The data were analyzed by two-way repeated measurement ANOVA followed by Tukey's post hoc test. A p-value <0.05 was considered significant.

**Results:**

NTS electrical waves did not change during ischemia time, while they significantly decreased during the entire reperfusion time. NTS electrical activity and BRS dramatically reduced in rats with I/R injury; however, administration of NAR, TMZ alone or their combination significantly improved these changes in rats with I/R injury.

**Conclusions:**

The results showed that I/R injury leads to reduced BRS and NTS electrical activity and there may be an association between I/R and decreased BRS. In addition, NAR and TMZ are promising agents to treat I/R complications.

## Introduction

Acute kidney injury (AKI) is a major clinical problem with high prevalence that affects more than 50% of patients in the intensive care unit (ICU) and causes mortality > 60%.^1^^,^^2^ Renal ischemia/reperfusion (I/R) is one of the most important causes of AKI and the generation of reactive oxygen species (ROS) play an important role in I/R injury events.^3^

The overproduction of free radicals in the I/R injury induces apoptosis and, ultimately, cell death and organ dysfunction.^3^ Oxidative stress (reactive oxygen species over the antioxidant defense system) is known as a factor in I/R injury.^3^ Free oxygen radicals and ROS are transmitted through the bloodstream to distant organs and are considered as intermediate agents of damage to distant organs resulting from I/R.^4^^,^^5^

The Nucleus tractus solitarius (NTS) acts as the gateway to the central nervous system for sensory information entry, which plays an important role in cardiovascular regulation.^6^ Peripheral baroreceptors, chemoreceptors and renal sympathetic afferent nerves create the primary synapse in the NTS.^7^ Baroreceptor dysfunction leads to loss of regulation of blood pressure fluctuations and impaired baroreceptor reflex sensitivity (BRS), which is a well-known pathophysiological basis of cardiovascular disturbances.^8^ Available evidence indicates that NTS degradation leads to blood pressure alternations.^7^ Therefore, NTS is one of the main centers for BRS regulation.^8^

Naringin (4, 5, 7-trihydroxyflavanone-7-rhamnoglucoside, NAR) is a polyphenol compound, which is found mainly in grapefruit and a number of citrus plants. Antimicrobial, anti-mutagenic, anticancer, anti-inflammatory, free radical scavenging and antioxidant effects of NAR have been shown.^9^^,^^10^ The protective effects of NAR through the increased activity of antioxidant enzymes have been documented.^9^^,^^10^

*Trimetazidine* (1-[2, 3, 4-trimethoxybenzyl] piperazine dihydrochloride, TMZ) is an anti-ischemic drug used in unstable angina.^11^ The mitochondrial permeability transition pore (mPTP), located on the inner membrane of mitochondria plays a powerful role in the production of ROS and the onset of apoptosis following I/R injury.^12^ An experimental study documented that TMZ was able to inhibit mPTP and reduce the infarction size in myocardial I/R injury and caspase-3 activity.^13^ Moreover, the inhibition of lipid peroxidation by TMZ has been reported.^14^ The aim of this study was to evaluate the effect of NAR and TMZ alone and as a combined therapy on the local field electrical activity of the NTS at the local electroencephalography (EEG) and BRS sensitivity following renal I/R injury.

## Methods

### Drugs

TMZ, NAR, ketamine, xylazine and urethane were purchased from Sigma Co. USA. NAR and TMZ were dissolved in distilled water and urethane was dissolved in normal saline solution just before use.

### Animals

In current study, and according to our previous study and other similar ones, forty male Sprague-Dawley rats (weighing 200- 250 g) were purchased from an animal breeding and care center of Ahvaz Jundishapur University of Medical Sciences (AJUMS). All animals were housed in standard cages (4 in each cage) under controlled temperature (22 ± 2 °C), humidity (50-55%) and a 12-h light/dark cycle (lights on at 07:00 am), with free access to food chow pellets and tap water. The rats were simply and non-randomly allocated into five groups of eight animals in each. Groups: 1) Sham, 2) I/R, 3) TMZ 5 mg/kg; I/R rats received TMZ (5 mg/kg, iv) five minutes before reperfusion,^15^ 4) NAR 100 mg/kg; I/R rats received NAR (100 mg/kg, i.p) once daily for seven days before I/R,^10^ and 5) TMZ 5 mg/kg + NAR 100 mg/kg; I/R rats received TMZ 5 mg/kg + NAR 100 mg/kg. Rats in sham and I/R groups received vehicle (sterile saline solution). Rats in the sham group were submitted to the same surgical procedure as the other groups, without the use of clamps and I/R induction.

### Stereotaxic surgery to implant electrode

A week prior to EEG recording, the rats were anesthetized with ketamine (50 mg/kg) and xylazine (5 mg/kg), intraperitoneally. The rats’ body temperature was maintained at 36.5±0.5 ° C using heating pads, with their heads mounted in a stereotaxic device (Narishige Co. Japan) for electrode implantation surgery. A coated stainless steel Teflon bipolar metal wire electrode (0.005” bare, 0.008” coated, A-M systems, Inc. WA) was implanted in the NTS with Paxinos and Watson stereotaxic atlas, with coordination of AP=-14.04 mm from bregma; ML= 0.4 mm, and DV=8 mm from the dura, correspondingly.^16^ All implants were fixed to the skull using dental acrylic cement and two small glass anchor bolts.

### Induction of renal ischemia/reperfusion (I/R)

Rats were kept in fasting condition overnight prior to the surgery (for at least 10 h) but had free access to water. At the day of surgery, the rats from each group were anaesthetized with urethane (1.7 g/kg, i.p).^17^ Then, the rats were placed on heating pads (Harvard Apparatus, UK) to keep the body temperature approximately at 37 ° C. Fifteen minutes after the anesthesia, the left femoral vein was catheterized using a polyethylene catheter (PE50) to infuse saline solution or TMZ and the left femoral artery was used to measure blood pressure and baroreflex sensitivity. The left and right kidneys were exposed through a midline incision. Bilateral ischemia was induced by occluding both renal pedicles using non-traumatic clamps for 45 min. After that, the clamps were removed and reperfusion continued for 4 hours.^18^

### Local EEG recording

The local field potentials (local EEG) from the rats’ NTS were fed to a ML135 bio amplifier (4-Channels data acquisition Power Lab. and Lab Chart software version 7, AD Instruments Co., Australia) with 1 mV amplification, 400 Hz sampling rate, and 0.3–70 Hz band pass filtration for 5 minutes. The basic 5-sec EEG variation period were compared in all groups. The electrical power of frequency bands were measured as mV^2^/Hz. The local EEG recording was performed before ischemia for 45 min, during ischemia and reperfusion time, correspondingly.

### Mean arterial pressure measurement

The mean arterial pressure was recorded through the catheterization of the left femoral artery that connected to a pressure transducer and monitored by a Power Lab System (AD Instruments, Australia), before ischemia for 20 min for adaptation, during ischemia and reperfusion time.

### Baroreflex sensitivity

In all groups, at the end of reperfusion period, intravenous injections of phenylephrine (10 to 20 µg/kg) were performed and the changes in blood pressure and heart rate were recorded using pressure transducer and monitored and recorded on PC, using Lab Chart software. There were 15 minute-intervals for recovery between drug injections to reach the previous level of blood pressure. For each injection, the maximum amplitude of the resulting pressure and bradycardia were used to calculate the mean arterial pressure (ΔMAP) and heart rate changes (ΔHR). The ratio of ΔHR change to ΔMAP change was used as the BRS index.^19^

### Statistical analysis

The data obtained for mean arterial pressure, heart rate and local EEG were analyzed with two-way repeated measurement ANOVA followed by Tukey's test as a post hoc test for multiple comparisons using Prism software, version 6.0 (San Diego, CA). The data were expressed as the mean and standard deviation (SD). P-values < 0.05 were considered as significant differences.

## Results

### The effect of NAR, TMZ or their combination on NTS local field electrical activity

There were no significant alterations in NTS electrical power during pre-I/R and 45-minute ischemia period. However, NTS electrical power was dramatically decreased during the entire 1^st^, 2^nd^, 3^rd^ and 4^th^ hours of reperfusion period in the I/R group compared with sham rats. On the other hand, the administration of NAR and TMZ alone or in combination improved its power compared with the I/R group (Table 1).

**Table 1:** Effect of Naringin (NAR), Trimetazidine (TMZ) or their combination pretreatment on NTS electrical activity following renal I/R injury

Groups	EEG Power (mV2/Hz)					
	Pre-IR	Ischemia	Reperfusion Time (h)			
			1st	2nd	3rd	4th
Sham	0.723 ± 0.117	0.675 ± 0.126	0.699 ± 0.116	0.68 ± 0.104	0.673 ± 0.104	0.695 ± 0.13
IR	0.725 ± 0.081	0.652 ± 0.052	0.635 ± 0.095***	0.61 ± 0.082***	0.587 ± 0.042***	0.592 ± 0.025***
NAR	0.705 ± 0.034	0.653 ± 0.051	0.673 ± 0.029	0.65 ± 0.033#	0.63 ± 0.041#	0.646 ± 0.038##
TMZ	0.712 ± 0.067	0.668 ± 0.085	0.673 ± 0.073	0.65 ± 0.069#	0.632 ± 0.054#	0.637 ± 0.069#
NAR+TMZ	0.75 ± 0.07	0.65 ± 0.069	0.673 ± 0.064	0.65 ± 0.047#	0.627 ± 0.049#	0.64 ± 0.053##

Data were represented as mean ± SD (n=8). Sham (sham operated group), I/R (Ischemia-reperfusion + normal saline), NAR (I/R + NAR 100 mg/kg, i.p, for one week), TMZ (I/R + TMZ 5 mg/kg, iv, before reperfusion). NAR + TMZ, combination of NAR and TMZ. Repeated measure-Two-way ANOVA followed by Tukey's post hoc test.

*** p<0.001, vs. sham group.

# p<0.05,

## p<0.01, vs. I/R group.

### Effect of NAR and TMZ on heart rate and arterial pressure

Renal I/R injury significantly reduced the heart rate, while pretreatment with NAR, TMZ or their combination somewhat restored the heart rate to the normal values (Figure 1). Regarding the mean arterial pressure, the results showed no differences between the different groups (Figure 2).

**Figure 1:** Effect of Naringin (NAR), Trimetazidine (TMZ) or their combination pretreatment on heart rate, following renal I/R (I/R). Data were represented as mean ± SD (n=8). Sham (sham operated group), I/R (Ischemia-reperfusion + normal saline), NAR (I/R + NAR 100 mg/kg, i.p, for one week), TMZ (I/R + TMZ 5 mg/kg, iv, before reperfusion). NAR + TMZ combination of NAR and TMZ. Repeated measure-Two-way ANOVA followed by Tukey's post hoc test. *p<0.05, vs. sham group.

**Figure 2:** Effect of Naringin (NAR), Trimetazidine (TMZ) or their combination pretreatment on mean arterial pressure following renal I/R (I/R). Data were represented as mean ± SD (n=8). Sham (sham operated group), I/R (Ischemia-reperfusion + normal saline), NAR (I/R + NAR 100 mg/kg, i.p, for one week), TMZ (I/R + TMZ 5 mg/kg, iv, before reperfusion). NAR + TMZ, combination of NAR and TMZ. Repeated measure-Two-way ANOVA followed by Tukey's post hoc test.

### Effect of NAR and TMZ on BRS

As shown in figure 3, BRS was significantly reduced in the I/R injury group when compared to the sham group. However, the administration of NAR or TMZ restored it; so there was no difference between the sham, NAR and TMZ groups; however, their combination increased the BRS more significantly.

**Figure 3:** Effect of Naringin (NAR), Trimetazidine (TMZ) or their combination pretreatment on baroreceptor sensitivity (ΔHR/ΔMAP)) following renal I/R (I/R). Data were represented as mean ± SD (n=8). Sham (sham operated group), I/R (Ischemia-reperfusion + normal saline), NAR (I/R + NAR100 mg/kg, i.p, for one week), TMZ (I/R + TMZ 5 mg/kg, iv, before reperfusion). NAR + TMZ, combination of NAR and TMZ. Repeated measure-Two-way ANOVA followed by Tukey's post hoc test. *p<0.05, ***p<0.001, vs. sham group, #p< 0.05 vs. I/R group.

## Discussion

The findings of the current study demonstrated that the I/R injury weakened NTS electrical activity and BRS. However, changes in brain waves after I/R showed that NTS electrical activity was suppressed and these changes were affected by possible heart and kidney dysfunction. Subsequent abnormal NTS electrical activity can further impair cardiac function and aggravate ischemic complications in renal function. On the other hand, pre-treatment with NAR and TMZ alone restored gamma and delta electrical powers, while its combination with TMZ improved all other NTS recorded electrical waves and also restored BRS. The afferent inputs of baroreceptors are primarily received in the NTS, which has a neuronal complex relationship with other areas of the central nervous system and the vasomotor area (the ambiguous core and rostral ventrolateral medulla).^20^ Various studies have shown that diseases such as diabetes, hypertension, and renal failure mediated by oxidative stress can weaken the BRS.^21^ Acute kidney injury (AKI) increases the production of anti-inflammatory cytokines, and reduces the clearance of cytokines, thus leading to increased systemic inflammatory responses.^22^^,^^23(19^^,^^20)^ A previous study has shown a relative correlation between baroreceptor dysfunction and oxidative stress.^24^ Other studies showed that antioxidants can improve BRS in various experimental models.^25^ On the other hand, it was found that the administration of free radical scavengers, such as superoxide dismutase (SOD) and catalase (CAT) in rabbits suffering from atherosclerosis was able to improve baroreceptor function, indicating the inhibition of ROS effect on the baroreceptor performance.^26^

In the present study, NAR and TMZ alone or in combination improved baroreceptor sensitivity, which may have occurred due to their antioxidant effects through lipid peroxidation scavenging. Our previous study has shown that NAR, TMZ or their combination can reduce glomerular dysfunction by enhancing antioxidant capacity and reducing the microRNA-10a level.^27^ In line with this study and other previous findings, BRS is attenuated by oxidative stress, and polyphenol compounds improve it by scavenging free radicals.^28^ In addition, extensive evidence indicated that I/R led to the reduction of nitric oxide synthesis, which is a major contributor of endothelial dysfunction, followed by baroreceptor dysfunction.^29^ In this regard, NAR improves endothelial dysfunction by synthesizing and increasing nitric oxide bioavailability.^30^ In a human study, it has also been shown that TMZ improved endothelial dysfunction in chronic heart failure by reducing lipid peroxidation levels.^31^ An experimental study showed that TMZ reduced malondialdehyde, a renal oxidative injury index in a renal I/R injury model.^14^ TMZ stimulates glucose oxidation by reducing the oxidation of beta-fatty acids, which leads to the production of ATP with less oxygen consumption.^11^

The results of this study showed BRS improvement with NAR and TMZ pretreatment in I/R. Although the precise mechanism of the antioxidant effects of NAR and TMZ on BRS is unclear in renal I/R, it is possible that NAR, TMZ or their combination increase BRS in renal I/R by improving the autonomic nervous system function. Recently, it has been shown that the administration of antioxidants can improve BRS by improving autonomic function.^24^ The central mechanisms of NAR and TMZ need to be clarified in further studies on the autonomic nervous system function.^25^

The reduction in renal function leads to the accumulation of toxins and increased serum osmolality, which can directly stimulate vascular endothelial growth factor synthesis. In addition, the increase in ROS production leads to endothelial damage and permeability of the blood-brain barrier (BBB).^32^^,^^33^ Several lines of studies have shown that BBB performance was disrupted in experimental models of AKI indicated by Evans blue dye over the permeability into the brain tissue.^34^^,^^35^ On the other hand, experimental inflammatory models induced by alpha tumor necrosis factor (TNF-α) abnormally increases the permeability of the BBB. These experimental studies support the idea that inflammation associated with AKI elevates the inflammatory cytokines in the bloodstream and this can impair BBB permeability.^36^ Extensive evidence showed that NAR is a potent antioxidant that crosses the BBB and reduces inflammatory factors to protect the brain.^37^^,^^38^ A sudden drop in kidney function leads to toxin accumulation and increased serum osmolality, which can increase ROS, resulting in endothelial injury, BBB and brain transmitter disruption.^39^

An experimental study showed that cerebral I/R injury results in changes in cardiac electrophysiological parameters, as well as reduction in NTS electrical activity. However, the administration of anti-oxidants prevented these complications.^16^ The reduction in oxygen availability to the neuronal system, followed by the ruptured blood vessels, led to a cascade of events, including activation of glutamate receptors and Ca^2+^ influx.^40^ The activation of glutamate receptors caused an increase in cytoplasmic Ca^2+^ concentrations, as the result of Ca^2+^ influx through α-amino-3-hydroxy-5-methylisoxazole- 4-propionate (AMPA) and *N*-methyl-D-aspartate (NMDA) receptor channels, and voltage-dependent Ca^2+^ channels (VDCC). The opening of inotropic glutamate receptors, and the consequent influx of Na^+^ and Ca^2+^, is identified as the first stages in the excitotoxic process.^40^

Another experimental study showed that AKI induced by renal I/R led to renal dysfunction and increased renal sympathetic nerve activity and increased norepinephrine concentrations, indicating the role of the sympathetic nervous system in the development of AKI.^41^ Recently, the role of renal nerves in the renal I/R model showed that renal sympathetic nerve denervation improved renal function, reduced the response of inflammatory factors and apoptosis without changes in blood pressure.^42^ While Mitaka et al. showed that renal I/R led to a reduction in blood pressure and no changes in heart rate, which is opposed to the results of this study.^43^ This controversy may be related to the experimental model, the animal species and the reperfusion period. Our previous study indicated that renal I/R injury result in kidney dysfunction and myocardial injury and pre-treatment with NAR and TMZ alone or their combination might have a protective role on the remote effect of AKI on oxidative stress and myocardial injury through Nrf-2 regulation.^44^

### Limitations

This study had some limitations. Firstly because it is part of a Ph.D. thesis, including financial and time limitations. Therefore, we could not identify some parameters such as brain histology and measurement of molecular and antioxidant parameters in the brain tissue. Our aim was to investigate BRS and NTS activity in the AKI model.

## Conclusion

Our findings, along with other research findings, are in line with the present study, which suggest the reduction in renal function due to renal I/R injury, leading to a reduction in BRS and NTS electrical activity. Probably, there is an association between renal function reduction and BRS decrease, although NAR and TMZ alone or their combination improved the BRS and the NTS electrical activity. However, it can be hoped that these antioxidant agents can be used to prevent renal I/R complications in areas beyond the injury site.
